# Associations between systemic inflammatory markers and cognitive decline in patients with early-stage Alzheimer’s disease: a retrospective clinical study

**DOI:** 10.3389/fneur.2026.1765287

**Published:** 2026-03-18

**Authors:** Yunyang Liu, Yashuang Li, Pengbin Zheng, Bingjie Zhang

**Affiliations:** Department of Neurosurgery, Tianjin First Central Hospital, Tianjin, China

**Keywords:** cognitive decline, early-stage Alzheimer’s disease, Interleukin-6, retrospective study, systemic inflammatory markers, tumor necrosis factor-alpha

## Abstract

**Background:**

Emerging evidence implicates systemic inflammation in Alzheimer’s disease (AD)’s development and progression, yet comprehensive clinical data linking specific systemic inflammatory biomarkers to cognitive decline in early-stage AD remain limited.

**Objective:**

To evaluate the correlation between key systemic inflammatory biomarkers and cognitive decline in early-stage AD, to identify potential inflammatory indicators for risk screening and disease monitoring.

**Methods:**

In this retrospective study, 155 patients with early-stage AD and 100 matched healthy controls were enrolled between March 2020 and March 2025. Peripheral blood levels of inflammatory biomarkers, including IL-1β, IL-6, IL-8, IL-10, TNF-α, MCP-1, and CRP, were measured. Cognitive function was assessed using the MMSE and MoCA. Group comparisons, Spearman correlation analyses, and ROC curves with DeLong tests were performed.

**Results:**

The groups were comparable in baseline demographics (*p* > 0.05). The AD group exhibited significantly lower MMSE and MoCA scores (*p* < 0.001). AD patients had significantly elevated plasma levels of IL-1β, IL-6, TNF-α, and MCP-1 (*p* < 0.001), and decreased levels of IL-8 and IL-10 (*p* < 0.001). Correlation analyses revealed significant negative correlations between MMSE/MoCA scores and IL-1β, IL-6, TNF-α, and MCP-1 (*p* < 0.05), and positive correlations with IL-8 and IL-10 (*p* < 0.05). IL-6, IL-1β, and TNF-α showed the strongest associations. ROC analysis indicated AUCs of 0.766 for IL-1β, 0.716 for TNF-α, and 0.768 for IL-6. A panel combining IL-1β, TNF-α, and IL-6 achieved a significantly higher AUC of 0.894, with 77.42% sensitivity and 86.00% specificity.

**Conclusion:**

Elevated levels of IL-6, IL-1β, and TNF-α are strongly associated with cognitive decline in early-stage AD, suggesting their utility as potential biomarkers for disease progression. A multi-marker inflammatory panel significantly enhances diagnostic accuracy, supporting the exploration of anti-inflammatory strategies for early intervention.

## 1 Introduction

Alzheimer’s disease (AD) has an insidious onset, with its early clinical manifestations primarily characterized by short-term memory impairment (1). Patients often exhibit typical symptoms such as forgetting recent events or repeating questions. As the disease progresses, memory deficits gradually extend to the long-term memory system, leading to an inability to recall significant personal experiences or recognize family and friends, which severely impacts social functioning (2). Beyond memory impairment, AD patients may also experience a decline in language function, disorientation, behavioral abnormalities, and various neuropsychiatric symptoms (3). In the advanced stages of the disease, patients suffer from comprehensive cognitive and functional deterioration, ultimately losing the capacity for basic activities of daily living and requiring full-time care (4). According to the WHO, 50 million people worldwide live with dementia, a number projected to exceed 152 million by 2050 due to population aging (5). This trajectory poses a significant challenge to aging societies globally and places a heavy social and economic burden on public health systems worldwide.

AD is defined neuropathologically by two lesions: extracellular clusters of β-amyloid (Aβ) forming plaques, and intracellular bundles of hyperphosphorylated tau forming tangles (6). These two pathological alterations are widely recognized as the hallmark lesions of AD and hold a pivotal position in disease diagnosis (7). With ongoing research advancements, recent evidence indicates that the pathogenesis of AD also involves the interplay of multiple biological processes and signaling pathways. These include neuroinflammation, oxidative stress, aberrant lipid metabolism, mitochondrial dysfunction, the microbiota-gut-brain axis, and impaired autophagy (8). Among these, neuroinflammation is a critical contributor to AD (9). Some scholars posit that sustained inflammatory immune responses in the brain have emerged as the third core pathological feature, following Aβ deposition and neurofibrillary tangle formation (10). Genome-wide association studies (GWASs) link genes encoding immune receptors to the onset and progression of AD (11). Furthermore, recent research has identified genes involved in regulating immune function as AD risk genes (12).

Postmortem studies of AD patients have consistently demonstrated the presence of neuroinflammatory responses, further confirming its significance in AD pathogenesis (13). Research indicates that Aβ deposition triggers a cascade of microglia-mediated neuroinflammatory responses. Activation of the NF-κB, p38 MAPK, caspase, nitric oxide, and cyclooxygenase pathways stimulates cerebral immune cells to release inflammatory cytokines like ILs, TNF, and IFN, which actively contribute to AD pathology (14). Synaptic failure, neuronal loss, and suppressed neural activity are driven by the surge of pro-inflammatory cytokines, whereas anti-inflammatory cytokines can suppress neuroinflammation and reduce neuronal damage, thereby protecting cognitive function (15). A decline in the function of the anti-inflammatory system may exacerbate neuroinflammation. Consequently, pharmacological agents with anti-neuroinflammatory activity have garnered significant attention as potential candidate therapies for AD.

Despite its prominence, the inflammation hypothesis lacks consistent evidence linking systemic markers to cognitive decline in early-stage AD. Crucially, most studies have not specifically focused on the early stages of the disease process, a potential window of reversibility or modifiability for neuropathological changes, where targeted interventions might most effectively slow progression. Whereas sampling CSF biomarkers requires an invasive lumbar puncture that is difficult to scale, blood-based biomarkers are less invasive and easier to implement in both trials and clinical practice (16). Therefore, exploring hematological inflammatory markers is of great importance for the diagnosis and treatment of early AD.

This study explores whether inflammatory markers predict cognitive deterioration in incipient AD; we expect selected pro-inflammatory cytokines to rise in tandem with poorer neuropsychological performance, while certain anti-inflammatory cytokines may demonstrate protective associations. To test this, we enrolled rigorously diagnosed early AD patients and demographically matched healthy controls, measuring a range of inflammatory markers in their peripheral blood. By comparing marker levels between groups and correlating them with MMSE and MoCA scores, we will identify the inflammatory indicators most closely associated with cognitive decline in early AD. This study aimed to address existing gaps by employing a high-sensitivity multiplex assay to evaluate a broad panel of systemic inflammatory markers in a rigorously diagnosed early-stage AD cohort, with a particular focus on identifying a multi-marker signature that could enhance diagnostic accuracy in a Chinese clinical population.

## 2 Methods

### 2.1 Subjects

**Figure 1:**
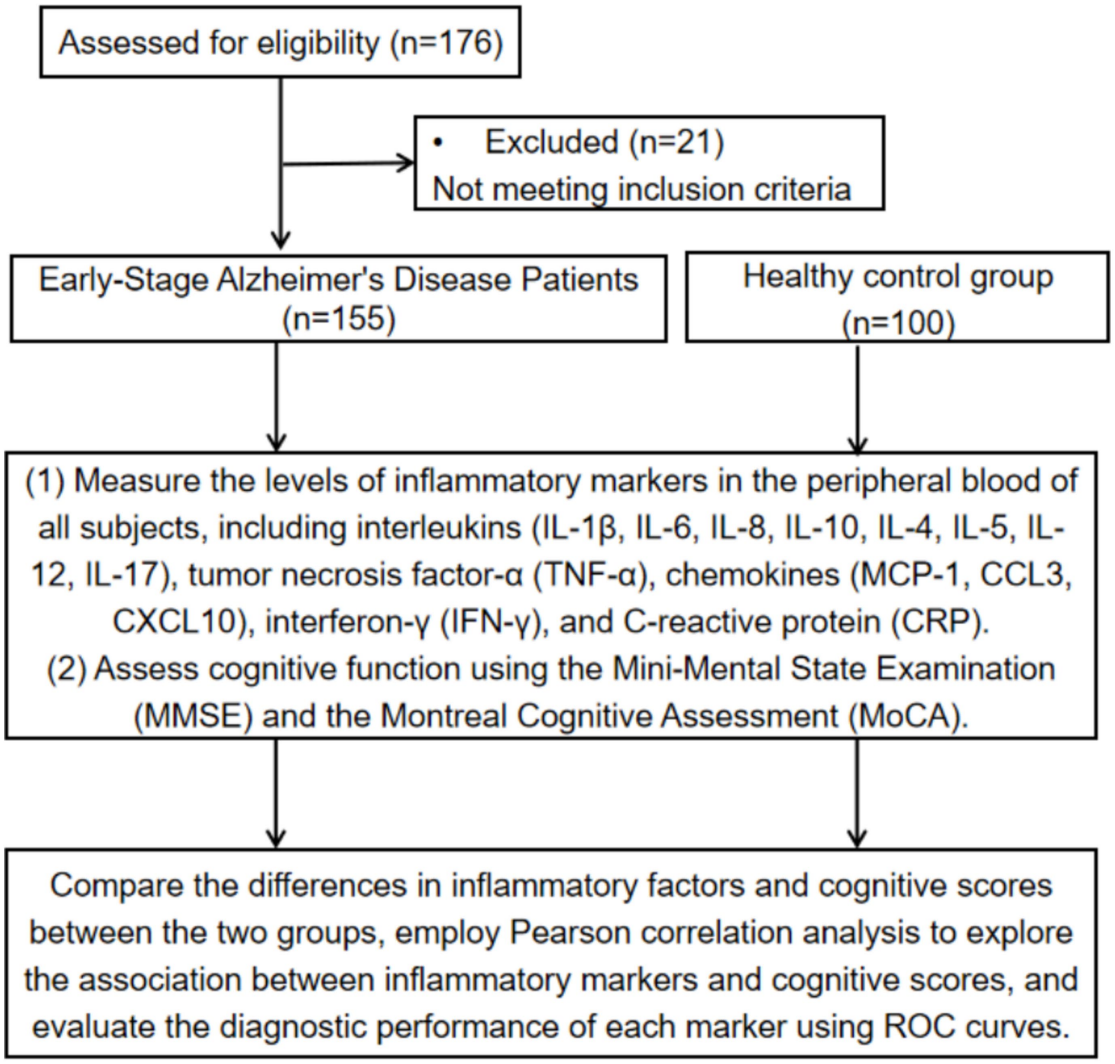
Research process.

This study selected 155 patients with early-stage AD group diagnosed in the Department of Neurology of our hospital between March 2020 and March 2025. Additionally, 100 healthy individuals who underwent physical examinations during the same period, matched for age and sex, were recruited as a healthy control (HC) group. All early-stage AD patients were jointly diagnosed by two experienced neurologists in accordance with established international diagnostic criteria. The study protocol was reviewed and approved by the Hospital Ethics Committee and conformed to the principles of the Declaration of Helsinki. Due to the retrospective nature of the research, the Ethics Committee granted a waiver for informed consent. The study flowchart is presented in Figure 1.

### 2.2 Diagnostic criteria for early-stage AD

(1) Core manifestations of episodic memory impairment, which may be accompanied by deficits in language, executive function, or visuospatial skills. Cognitive decline must have persisted for over 6 months, and patients should retain the ability to perform basic activities of daily living independently.
(2) Cerebrospinal fluid (CSF) analysis revealing a decreased Aβ42/Aβ40 ratio, indicative of cerebral Aβ plaque deposition, alongside elevated p-tau and t-tau, suggesting the presence of neurofibrillary tangles and neuronal injury.
(3) Positive findings on biomarker imaging, including amyloid PET tracer retention consistent with cerebral Aβ deposition, and structural MRI demonstrating hippocampal volume loss or medial temporal lobe atrophy.
(4) Exclusion of other potentially reversible causes, such as cerebrovascular disease, metabolic disturbances, medication effects, or depression, by experienced neurologists based on comprehensive history-taking, physical examination, and laboratory investigations (17).

### 2.3 Inclusion criteria

(1) AD group: Diagnosis of early-stage AD confirmed by at least two attending neurologists. All patients were seeking medical attention for the first time and had not received any prior AD-specific pharmacologic or other treatments. Availability of complete clinical data, cognitive assessment results, and peripheral blood inflammatory marker profiles.
(2) HC group: Underwent health check-ups at our hospital’s Health Management Center during the same period as the AD group recruitment. Matched to the AD group in terms of age and sex. No subjective complaints of memory decline or other cognitive impairment. No significant deficits in occupational, social, or daily living activities. Availability of complete clinical data, cognitive assessment results, and peripheral blood inflammatory marker profiles.

### 2.4 Exclusion criteria

(1) AD group: History of sudden onset of symptoms. History of major psychiatric disorders and other types of dementia. History of significant traumatic brain injury. History of severe cerebrovascular disease. History of substance abuse or chronic alcoholism. Recent major systemic illness (e.g., severe active infection, malignant tumor). Inability to cooperate with cognitive assessments due to aphasia, severe hearing/visual impairment, or other physical conditions. Recent use of medications known to influence inflammatory cytokine levels (e.g., antibiotics). Contraindications to MRI (e.g., claustrophobia, presence of magnetic-sensitive implants).
(2) HC group: Any personal history of definite neurological disorders or a family history (first-degree relatives) of early-onset dementia. Acute infection within 1 month prior to enrollment, or diagnosis of a chronic inflammatory disease requiring long-term therapy. Presence of other uncontrolled major medical conditions, or a history of stroke, intracranial space-occupying lesions, or other significant brain diseases.

### 2.5 Sample size calculation

Drawing on a moderate effect size (Cohen’s d = 0.4) reported in prior research (18), a retrospective power analysis was conducted. With *α* set at 0.05 (two-tailed) and power at 0.80, GPower 3.1 indicated a minimum of 100 participants per group for an independent-samples *t*-test. This study ultimately enrolled 155 early-stage AD patients and 100 healthy controls, resulting in a total sample size of 255 participants, thereby ensuring sufficient reliability and representativeness of the study findings.

### 2.6 Study methods

(1) Collection of clinical data: Basic information was collected for all participants through the electronic medical record system. This included age, sex, years of education, and history of underlying conditions such as hypertension and diabetes.
(2) Cognitive function assessment: All participants underwent standardized cognitive assessments. The MMSE and the MoCA were administered to comprehensively evaluate global cognitive function (19). The MMSE has a maximum score of 30 and primarily assesses orientation, memory, attention, calculation, and language abilities (20). The MoCA, which has a maximum score of 30, evaluates domains such as attention, memory, language, and executive functions. A score below 26 suggests cognitive impairment, a threshold adjusted by adding one point for individuals with fewer than 12 years of education (21).
(3) Measurement of inflammatory markers: A 5 mL fasting peripheral venous blood sample was collected from all participants on the morning of their hospital admission or health check-up. The levels of the following inflammatory markers were measured using Single Molecule Array (Simoa) technology (HD-1 analyzer, Quanterix, USA): Interleukins (IL-1β, IL-6, IL-8, IL-10, IL-4, IL-5, IL-12, IL-17), TNF-α, Chemokines (MCP-1, CCL3, CXCL10), IFN-γ, and CRP.

## 3 Statistical analysis

Data were analyzed with SPSS software (version 26.0). The normality of continuous variables was assessed using the Shapiro–Wilk test. Normally distributed data are presented as Mean ± Standard Deviation (SD) and compared between groups using the independent samples t-test. Non-normally distributed data are presented as Median with Interquartile Range (IQR) and compared using the Mann–Whitney U test. Categorical data are presented as frequencies and percentages [*n* (%)] and compared using the chi-square test. Associations between inflammatory marker levels and cognitive scores (MMSE, MoCA) were assessed using Spearman’s rank correlation coefficient. The discriminatory power of individual inflammatory markers and their combination for identifying early-stage AD was evaluated using Receiver Operating Characteristic (ROC) curve analysis, with the Area Under the Curve (AUC) reported. The DeLong test was used to compare the AUCs of different markers/panels. A two-tailed *p*-value < 0.05 was considered statistically significant.

## 4 Results

### 4.1 Baseline characteristics

The early AD and HC groups were comparable in baseline characteristics, with no significant differences in age, sex, BMI, education, marital status, number of children, or the prevalence of hypertension, diabetes, and hyperlipidemia (*p* > 0.05). See Table 1.

**Table 1:** Baseline data.

Variables	AD group (*n* = 155)	HC group (*n* = 100)	t/Z/*χ*^2^	*p*
Age, mean ± SD	65.46 ± 8.65	66.78 ± 9.22	−1.161	0.247
BMI, mean ± SD	23.45 ± 3.51	24.09 ± 3.13	−1.507	0.133
Years of education, median (IQR)	8.00 (3.00, 10.00)	8.00 (5.00, 11.00)	−1.315	0.189
Sex, *n* (%)			0.178	0.673
Female	92 (59.35)	62 (62.00)		
Male	63 (40.65)	38 (38.00)		
Spouse, *n* (%)			0.385	0.535
Yes	139 (89.68)	92 (92.00)		
No	16 (10.32)	8 (8.00)		
Children, *n* (%)			1.888	0.169
Yes	132 (85.16)	91 (91.00)		
No	23 (14.84)	9 (9.00)		
Hypertension, *n* (%)			0.531	0.466
Yes	43 (27.74)	32 (32.00)		
No	112 (72.26)	68 (68.00)		
Diabetes, *n* (%)			0.316	0.574
Yes	18 (11.61)	14 (14.00)		
No	137 (88.39)	86 (86.00)		
Hyperlipidemia, *n* (%)			0.164	0.686
Yes	19 (12.26)	14 (14.00)		
No	136 (87.74)	86 (86.00)		

BMI, body mass index. Normally distributed data are presented as mean ± SD; non-normally distributed data are presented as median (IQR).

### 4.2 Comparison of cognitive scores

**Figure 2:**
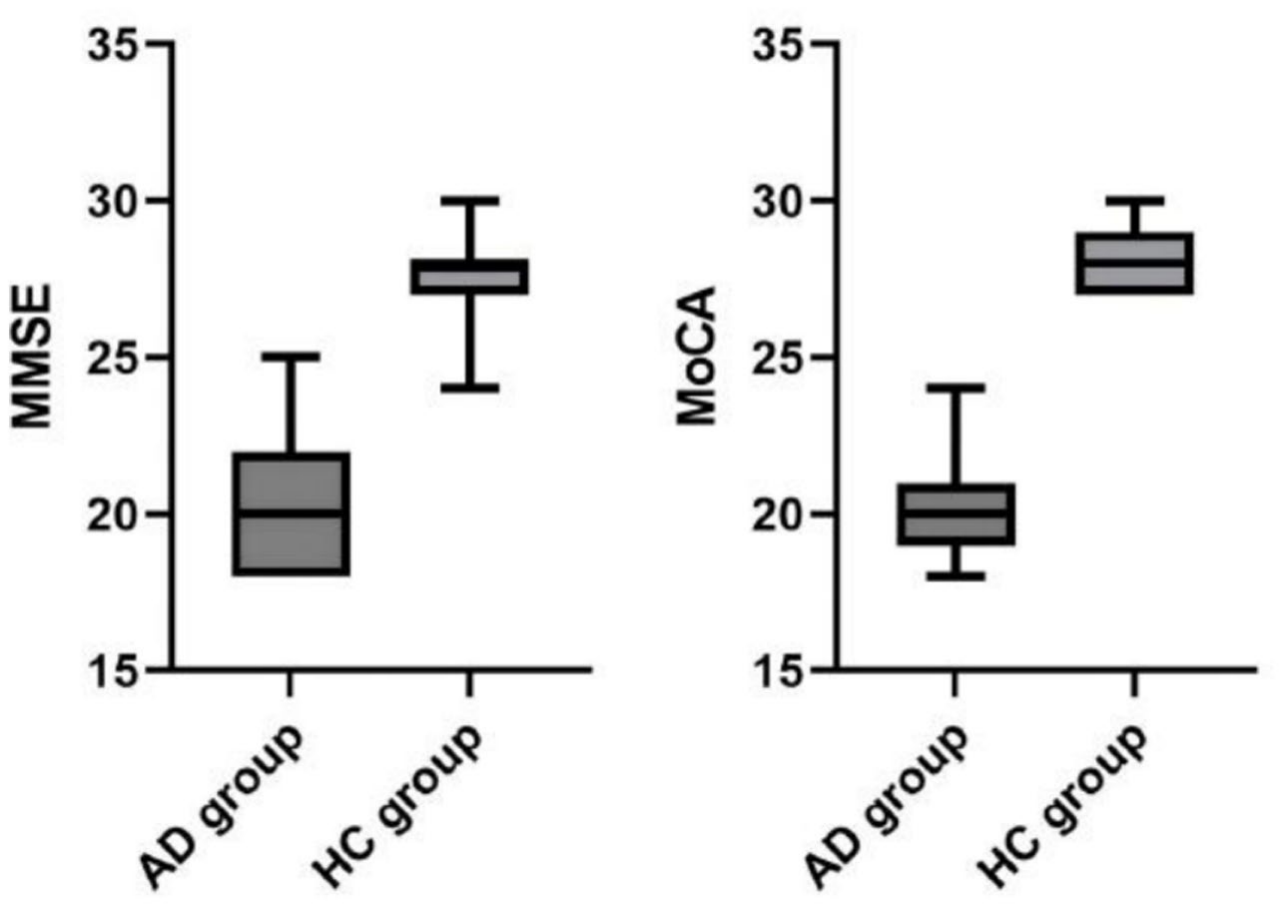
Comparison of cognitive scores.

Regarding cognitive function assessment, Table 2 shows that the median (interquartile range) MMSE and MoCA scores in the AD group were 20.00 (18.00, 22.00) and 20.00 (19.00, 21.00), respectively. These scores were significantly lower than those in the HC group, which were 28.00 (27.00, 28.00) for MMSE and 28.00 (27.00, 29.00) for MoCA (all *p* < 0.001). These results indicate significant overall cognitive impairment in patients with AD (see Figure 2).

**Table 2:** Comparison of cognitive scores [median (IQR)].

Group	*n*	MMSE	MoCA
AD group	155	20.00 (18.00, 22.00)	20.00 (19.00, 21.00)
HC group	100	28.00 (27.00, 28.00)	28.00 (27.00, 29.00)
*z*		−13.602	−13.579
*p*		<0.001	<0.001

Scores are presented as median (IQR) due to non-normal distribution.

### 4.3 Comparison of systemic inflammatory marker levels

Compared to HC, the early AD group showed significantly elevated pro-inflammatory markers (IL-1β, IL-6, TNF-α, MCP-1) and reduced anti-inflammatory markers (IL-8, IL-10), with all differences significant at *p* < 0.001 (Table 3). No other measured indicators (IL-4, IL-5, IL-12, IL-17, CCL3, CXCL10, IFN-γ, CRP) differed significantly between groups (all *p* > 0.05).

**Table 3:** Comparison of systemic inflammatory marker levels (pg/mL, mean ± SD).

Variables	AD group (*n* = 155)	HC group (*n* = 100)	*t*	*p*
IL-1β	1.55 ± 0.41	1.12 ± 0.39	8.116	<0.001
IL-6	2.45 ± 0.76	1.70 ± 0.62	8.627	<0.001
IL-8	1.75 ± 0.65	2.23 ± 0.74	−5.445	<0.001
IL-10	0.68 ± 0.31	0.82 ± 0.37	−3.220	<0.001
IL-4	5.15 ± 3.42	5.58 ± 3.81	−0.952	0.342
IL-5	1.44 ± 0.82	1.34 ± 0.94	0.895	0.372
IL-12	1.87 ± 1.06	1.81 ± 1.20	0.419	0.676
IL-17	6.02 ± 4.18	5.74 ± 3.97	0.528	0.598
TNF-α	1.25 ± 0.33	0.97 ± 0.39	6.228	<0.001
MCP-1	246.88 ± 91.52	209.17 ± 84.67	3.307	<0.001
CCL3	7.22 ± 3.58	6.73 ± 4.01	1.022	0.308
CXCL10	147.86 ± 81.86	132.98 ± 76.61	1.453	0.148
IFN-γ	4.25 ± 2.87	3.82 ± 2.96	1.153	0.250
CRP	3.21 ± 2.55	3.15 ± 2.10	0.184	0.854

All inflammatory markers were tested for normality. Those with normal distribution (e.g., IL-1β, IL-6, TNF-α) remain as mean ± SD.

### 4.4 Spearman correlation analysis

Based on the statistically significant results from the intergroup comparisons presented in Table 3, six inflammatory markers (IL-6, TNF-α, IL-1β, MCP-1, IL-8, and IL-10) were selected for Spearman correlation analysis with MMSE and MoCA scores. In patients with early AD, inflammatory markers showed distinct correlations with cognitive scores (MMSE/MoCA). IL-6, TNF-α, IL-1β, and MCP-1 were negatively correlated with cognition (all *p* < 0.05), whereas IL-8 and IL-10 were positively correlated. Notably, IL-6, IL-1β, and TNF-α (*r* > 0.3) demonstrated the strongest association with cognitive decline. Consequently, these three inflammatory factors were identified as potential biomarkers for further diagnostic efficacy evaluation (see Table 4).

**Table 4:** Spearman correlation analysis of the association between inflammatory marker levels and MMSE and MoCA scores.

Inflammatory markers	IL-6	TNF-α	IL-1β	MCP-1	IL-8	IL-10
MMSE-*r*	−0.405	−0.321	−0.387	−0.157	0.174	0.130
MMSE-*P*	0.000	0.000	0.000	0.012	0.005	0.038
MoCA-*r*	−0.408	−0.361	−0.404	−0.166	0.236	0.160
MoCA-*P*	0.000	0.000	0.000	0.008	0.000	0.010

### 4.5 ROC curve analysis

**Figure 3:**
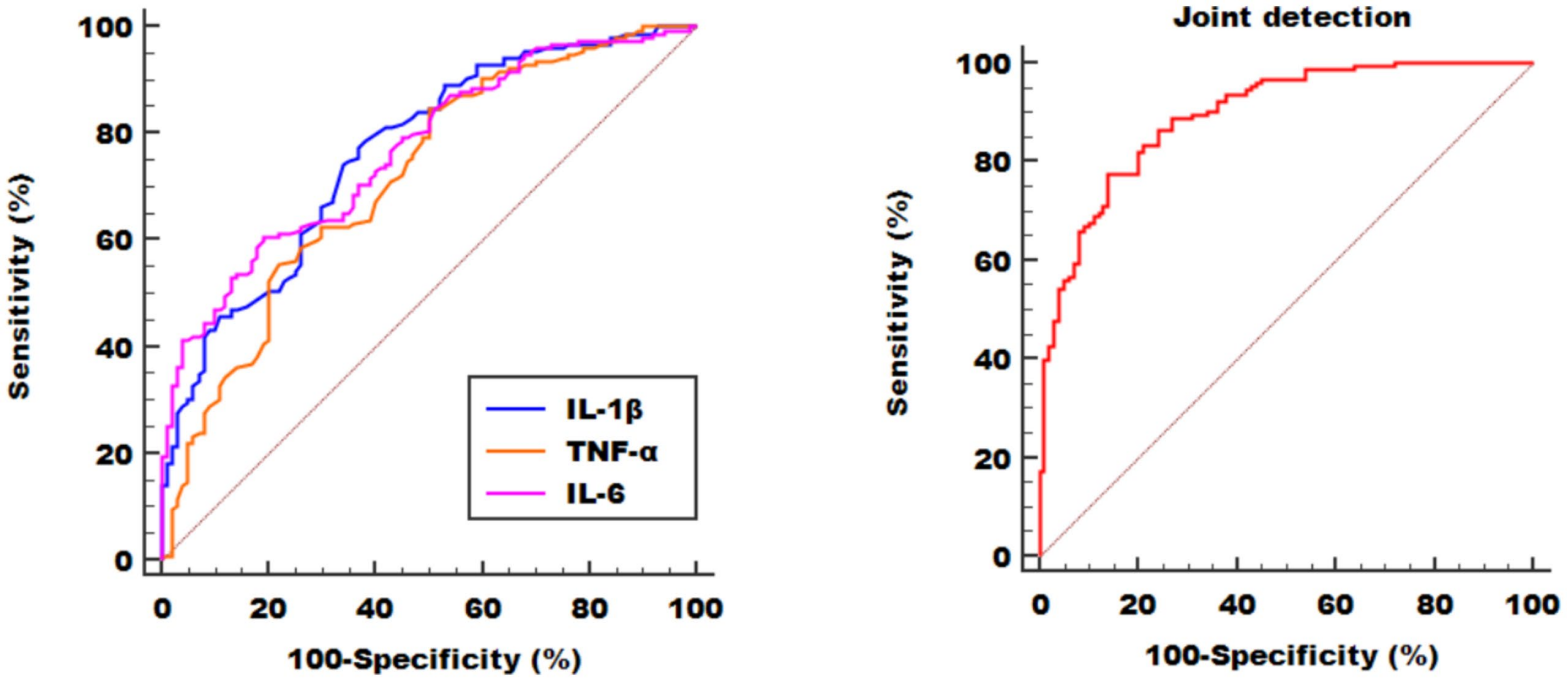
ROC curve of different indicators for predicting early-stage AD.

The results of the ROC curve analysis showed that the AUC for IL-1β, TNF-α, and IL-6 were 0.766, 0.716, and 0.768, respectively. Their optimal cutoff values were > 1.24 pg./mL, > 0.94 pg./mL, and > 2.19 pg./mL, corresponding to sensitivities of 78.71, 84.52, and 60.65%, and specificities of 62.00, 50.00, and 81.00%, respectively. The combined application of these three markers increased the AUC to 0.894, with a sensitivity of 77.42% and a specificity of 86.00%. This indicates that the combined panel significantly improves the ability to identify early-stage AD. See Table 5 and Figure 3.

**Table 5:** The AUC of different indicators.

Variables	Best cut-off value	Sensitivity	Specificity	AUC	SE^a^	95%CI^b^	*Z*	*p*
IL-1β	>1.24	78.71	62.00	0.766	0.030	0.709 ~ 0.816	8.880	<0.001
TNF-α	>0.94	84.52	50.00	0.716	0.033	0.657 ~ 0.771	6.526	<0.001
IL-6	>2.19	60.65	81.00	0.768	0.029	0.711 ~ 0.818	9.239	<0.001
Joint detection	—	77.42	86.00	0.894	0.020	0.850 ~ 0.929	19.970	<0.001

a DeLong et al. (1988)(37).

b Exact Binomial Test.

## 5 Discussion

Early identification and intervention for AD are crucial for reducing its global burden, and the role of systemic inflammation in AD pathogenesis has become a major research focus in recent years. Although growing evidence suggests that inflammatory cytokines contribute to neural damage in AD, clinical evidence systematically validating the quantitative association between specific systemic inflammatory markers and cognitive decline in early AD, their diagnostic efficacy, and underlying mechanisms remains scarce. This is particularly true for the clinical utility of blood-based biomarkers, where considerable controversy persists (22). Although prior studies have linked inflammation to AD, our work extends existing evidence by focusing exclusively on early-stage AD patients diagnosed via contemporary biomarker-supported criteria, utilizing high-sensitivity Simoa technology, and demonstrating the superior diagnostic performance of a combined inflammatory panel in a Chinese cohort. These elements strengthen the clinical relevance and novelty of our findings. Our research methodically analyzed associations between inflammatory biomarkers and mental decline in early-stage AD patients. The findings preliminarily support our initial hypothesis that a specific pattern of systemic inflammatory imbalance exists in early AD patients and that this imbalance is significantly correlated with the degree of cognitive impairment.

This study reveals markedly increased pro-inflammatory cytokines (IL-1β, IL-6, and TNF-α) in early-stage AD patients’ peripheral blood. These cytokines, particularly IL-6 and TNF-α, may impact the central nervous system through blood–brain barrier penetration or vagus nerve signaling, triggering microglial and astrocytic activation to drive neuroinflammation (23, 24). Two cross-sectional studies link peripheral inflammation to AD symptoms. Yasuno et al. (25) found that plasma IL-6 in AD patients correlated with the severity of behavioral and psychological symptoms (BPSD), suggesting a unique peripheral immune mechanism. Similarly, Sun et al. (26) reported that elevated plasma TNF-α levels in AD patients were negatively correlated with cognitive performance. However, the AUC for TNF-α alone as a diagnostic marker was 0.676, indicating low diagnostic performance compared to ATN biomarkers such as NfL, p-tau181, and Aβ42/Aβ40. Additionally, IL-1β, a key mediator of the early inflammatory response, can further amplify the expression of IL-6 and TNF-α, creating a positive feedback loop that exacerbates neuronal damage and synaptic dysfunction. Research by Park et al. (27) and a recent study by Remnitz et al. (28) found that IL-1β levels are significantly elevated in AD patients compared to those with mild cognitive impairment (MCI) and normal controls, and could serve as a potential inflammatory biomarker with an AUC of 0.953. Our findings are highly consistent with these studies, revealing strong negative correlations between these three cytokines and cognitive scores, suggesting their potential central role in early cognitive decline in AD.

The chemokine MCP-1 was markedly upregulated in AD patients. Studies consistently show that MCP-1, a chemokine responsible for monocyte/macrophage chemotaxis and activation, is elevated in the brain and blood of Alzheimer’s patients. For instance, Lee et al. (29) found that plasma MCP-1 levels were higher in AD patients than in MCI patients and controls, with the highest levels observed in severe AD patients. After adjusting for covariates using a generalized estimating equation, baseline MCP-1 levels were significantly associated with two-year changes in MMSE scores. MCP-1 may exacerbate the neuroinflammatory response by promoting the infiltration of inflammatory cells into the central nervous system, thereby contributing to the progression of both Aβ deposition and tau pathology (30). Our findings reinforce MCP-1’s involvement in AD-associated neuroinflammation, suggesting its potential as an early-phase biomarker.

Notably, plasma levels of IL-8 and IL-10, which were significantly lower in the early AD group, showed positive correlations with MMSE and MoCA scores. A meta-analysis by Shen et al. (31), encompassing 170 studies, similarly reported significantly lower peripheral blood IL-8 levels in patients with MCI compared to controls, although it did not mention a significant decrease in peripheral IL-10 in AD or MCI. Research by Porro et al. (32) suggested that decreased IL-10 expression in AD patients might increase individual susceptibility to the disease and be associated with an immunosuppressive state. Consistently, Pedrini et al.’s (33) blood biomarker analysis revealed a significant link between elevated IL-10 levels and amyloid pathology in cognitively normal subjects, highlighting its utility as a predictive indicator for AD susceptibility. IL-8, a chemokine, is known for its role in neutrophil chemotaxis. However, recent research indicates it may also possess neurotrophic and anti-inflammatory functions within the central nervous system (34). As a well-established anti-inflammatory cytokine, IL-10 protects neurons by mitigating neuroinflammatory responses and inhibiting pro-inflammatory factor release. The observed decrease in IL-8 and IL-10 levels in our study may reflect an impairment of anti-inflammatory mechanisms in AD patients, rendering them unable to effectively counter the persistent pro-inflammatory state. This imbalance likely contributes to further cognitive deterioration. In early AD, a heightened pro-inflammatory response alongside a dysfunctional anti-inflammatory system may significantly drive disease progression.

This study evaluated the diagnostic value of various inflammatory markers for early-stage AD using ROC curve analysis. The results showed that the AUC values for IL-1β, IL-6, and TNF-α were 0.766, 0.768, and 0.716, respectively, indicating moderate to good diagnostic performance in distinguishing early AD patients from healthy individuals. It is particularly noteworthy that the combined detection of these three markers increased the AUC to 0.894, with sensitivity and specificity reaching 77.42% and 86.00%, respectively, significantly outperforming any single marker. These findings suggest that a panel of multiple inflammatory markers may be more advantageous for the identification and screening of early AD, holding substantial potential for clinical translation. Compared to traditional AD biomarkers like CSF Aβ42 and p-tau, peripheral blood inflammatory marker testing offers advantages such as being non-invasive, easily repeatable, and lower in cost, making it more suitable for large-scale screening and long-term dynamic monitoring. Although the standardization and validation of blood biomarkers for AD diagnosis still face challenges, this study provides strong support for the application of inflammatory markers in the auxiliary diagnosis of early AD.

Our study observed no significant difference in CRP levels between the two groups, which contrasts with the findings of Tachibana et al. (35), potentially reflecting the limited sensitivity of CRP, as a non-specific acute-phase protein, in the early stages of AD, or its greater susceptibility to interference from other factors such as metabolism or concurrent infections. Our study found no significant differences in levels of IL-4, IL-5, IL-12, IL-17, or IFN-*γ* between the groups, suggesting that the roles of inflammatory factors in AD are specific. For instance, IL-17, a cytokine associated with Th17 cells, has a well-defined role in autoimmune diseases, but its part in AD remains controversial (36). The lack of a significant association with early AD found here might be related to the study population, disease stage, or methodological differences in detection.

The study’s outcomes carry notable clinical relevance. Inflammatory indicators such as IL-6, IL-1β, and TNF-α, strongly linked to cognitive deterioration in early-stage AD, provide new avenues for its early screening and risk assessment. Furthermore, measuring these inflammatory markers can help clinicians evaluate a patient’s immune-inflammatory status, thereby informing personalized treatment strategies. For instance, patients exhibiting a high inflammatory state might be more suitable for interventions involving drugs with anti-inflammatory properties. These results also provide clinical evidence from a Chinese population supporting the inflammatory hypothesis of AD, reinforcing the strategy of targeting inflammatory pathways for early intervention to delay disease progression. Future research should investigate the relationship between these inflammatory markers and neuroimaging or cerebrospinal fluid biomarkers, as well as their dynamic changes throughout the natural history of AD.

## 6 Study limitations

First, as a single-center retrospective study with a relatively limited sample size where all patients were recruited from a single institution, there is a potential for selection bias. Second, although confounding factors such as age, sex, and underlying diseases were controlled for, unmeasured confounders, such as dietary habits, lifestyle, and genetic background, might still influence the results. Third, this study only measured inflammatory marker levels at baseline and lacked longitudinal dynamic monitoring; therefore, a causal relationship with cognitive changes cannot be inferred. Although the detection method used for inflammatory markers in this study is highly sensitive, its standardization across different platforms requires further validation.

Furthermore, inflammatory marker levels and cognitive function were assessed only at a single time point. While this design can reveal correlations between variables, it cannot establish causality or temporal sequence between changes in inflammatory cytokine levels and cognitive decline. It remains unclear whether systemic inflammation drives AD pathology and cognitive impairment, whether AD neuropathological changes lead to secondary systemic inflammation, or whether both form a vicious cycle. Future prospective cohort studies with long-term, dynamic monitoring of patients are needed to clarify the evolutionary patterns and predictive value of inflammatory markers throughout the natural history of AD.

## 7 Conclusion

The findings implicate systemic inflammation in early-stage AD’s pathophysiology and cognitive decline, with peripheral inflammatory markers (IL-1β, IL-6, TNF-α) showing potential as diagnostic biomarkers. This evidence not only supports the inflammatory hypothesis but also suggests that anti-inflammatory interventions could be a promising therapeutic strategy.

## Data Availability

The original contributions presented in the study are included in the article/supplementary material, further inquiries can be directed to the corresponding author.
